# Bibliography

**Published:** 1921-02

**Authors:** 


### BIBLIOGRAPHY

### Lincoln Life Sketches

Under this title is just received a little book published by a dentist of many years' experience as a practitioner and writer on dental topics. He writes on a new subject but in just as entertaining a style as formerly, and so far as we can judge, what he has to say is just as entertaining and interesting as anything he formerly contributed. Any contribution to the literature of Abraham Lincoln will be read with great pleasure by every one, but when so good a friend and so able a scholar as Dr. Newkirk writes on such a subject we are assured that something will be said that needs to be said, and that it will be set forth in clear and attractive form, as has been done admirably in this case. We are greatly pleased with the new Lincoln stories and urge our readers to secure a copy of Dr. Newkirk's book, as it is worth while and it has a special appeal to anyone who may be interested in the literary character of the book. We haven't many dental authors but we should know those who have ventured beyond the technical and hope many will read this book. Published by Duffield & Co., 211 E. 19th St., New York City.

